# A Platform for the Glucose Biosensor Based on Dendritic Gold Nanostructures and Polyaniline-Gold Nanoparticles Nanocomposite

**DOI:** 10.3390/bios15030196

**Published:** 2025-03-19

**Authors:** Natalija German, Anton Popov, Arunas Ramanavicius, Almira Ramanaviciene

**Affiliations:** 1 Department of Immunology and Bioelectrochemistry, State Research Institute Centre for Innovative Medicine, Santariskiu 5, LT-08406 Vilnius, Lithuania; anton.popov@imcentras.lt; 2 NanoTechnas—Center of Nanotechnology and Materials Science, Faculty of Chemistry and Geosciences, Vilnius University, LT-03225 Vilnius, Lithuania; arunas.ramanavicius@chf.vu.lt

**Keywords:** dendritic gold nanostructures, glucose oxidase, gold nanoparticles, mediator-free biosensor, polyaniline

## Abstract

Diabetes mellitus is a pathological condition that requires continuous measurement of glucose concentration in human blood. In this study, two enzymatic mediator-free glucose biosensors based on premodified graphite rod (GR) electrodes were developed and compared. GR electrode modified with electrochemically synthesized dendritic gold nanostructures (DGNS), a cystamine (Cys) self-assembled monolayer (SAM), and glucose oxidase (GOx) (GR/DGNS/Cys/GOx) and GR electrode modified with DGNS, Cys SAM, enzymatically obtained polyaniline (PANI) nanocomposites with embedded 6 nm gold nanoparticles (AuNPs) and GOx (GR/DGNS/Cys/PANI-AuNPs-GOx/GOx) were investigated electrochemically. Biosensors based on GR/DGNS/Cys/GOx and GR/DGNS/Cys/PANI-AuNPs-GOx/GOx electrodes were characterized by a linear range (LR) of up to 1.0 mM of glucose, storage stability of over 71 days, sensitivity of 93.7 and 72.0 μA/(mM cm^2^), limit of detection (LOD) of 0.027 and 0.034 mM, reproducibility of 13.6 and 9.03%, and repeatability of 8.96 and 8.01%, respectively. The GR/DGNS/Cys/PANI-AuNPs-GOx/GOx electrode was proposed as more favorable for glucose concentration determination in serum due to its better stability and resistance to interfering electrochemically active species. The technological solutions presented in this paper are expected to enable the development of innovative mediator-free enzymatic glucose biosensors, offering advantages for clinical assays, particularly for controlling blood glucose concentration in individuals with diabetes.

## 1. Introduction

Diabetes mellitus is one of the most common causes of death, as well as acute and chronic disease (heart, retina, kidney failure, blindness, or limb amputations) worldwide [1,2,3], and it can be diagnosed by monitoring glucose in blood [4,5,6]. Type 1 diabetes affects approximately 20 million individuals [1] and raises the risk of hypoglycemia [3]. Glucose biosensors account for about 85% of the total biosensor market [2,3]. Enzymatic electrochemical biosensors for blood glucose monitoring have garnered significant interest owing to their multiple uses and cost-effectiveness, rapid response and accessibility, high sensitivity and exceptional selectivity, miniaturization capabilities, and long-term stability [1,2,3].

The application of electrochemical glucose biosensors based on enzymes (e.g., glucose oxidase (GOx), glucose dehydrogenase/laccase) has significantly increased due to the electrocatalytic activity and stability of enzymes [1,2,3,4,5,6,7,8]. Glucose is oxidized during the enzymatic reaction to glucono-1,5-lactone, whereas oxygen is reduced to hydrogen peroxide (H_2_O_2_), subsequently resulting in the formation of H_2_O [9,10,11,12]. The transfer of electrons from the GOx active center, which is deeply embedded within the protein shell, to the working electrode surface is hindered by the formation of an internal barrier, which makes direct electron transfer (DET) difficult [2,9]. This drawback can be solved by (i) using conducting compounds [12,13,14,15], (ii) employing redox mediators to shuttle electrons from the GOx redox center to the working electrode [6,16], or (iii) registering the consumption of oxygen (O_2_) or production of H_2_O_2_ [17,18]. The DET process in the mediator-free biosensors occurs between the FAD in the active center of the oxidized form of the enzyme (GOx(FAD)) and the surface of the working electrode involving two electrons for obtaining the reduced form of glucose oxidase (GOx(FADH_2_)) [11,12,19]. The two most important conditions for the DET process in mediator-free biosensors are (i) the orientation of the enzyme redox center towards the electrode and (ii) not more than 2 nm distance between the redox center of the enzyme and the electrode surface [20].

Advances in nanotechnology and nanobiotechnology have opened new opportunities in various fields, including bioanalytical chemistry, bioelectronics, biomedicine, pharmacology, agriculture, and environmental monitoring [2,5,10]. Generally, noble metal nanoparticles (e.g., gold nanoparticles (AuNPs)) [19,20,21,22,23,24,25], gold-coated magnetic iron oxide nanoparticles (Fe@Au) [26,27,28,29], gold nanostructures (DGNS) [30,31,32,33], gold nanorods [34] and nanocluster (AuNC) [18], nanoporous gold (NPAu) [13] and platinum nanoparticles (PtNPs) [35,36]), α-zirconium phosphate nanosheets [37], and carbon nanomaterials (e.g., carbon nanotubes [38,39], reduced graphene oxide–magnetic nanoparticles (RGO-Fe_3_O_4_) [11] and graphene [12]) are widespread as the platform for the construction of glucose [2,10], lactose [29], and lactate [31] biosensors. AuNPs are distinguished by their unique electronic, optical, and catalytic properties and are employed in the construction of biosensors due to their ability to wire the redox center of the enzyme with an electrode [15,16,19]. Gold nanoparticles improve the performance of glucose biosensors by increasing the surface area of the working electrode, facilitating charge transfer, and improving the immobilization of enzymes on the surface of the electrode [7,8].

Conducting polymers, such as polyaniline (PANI) and polypyrrole (Ppy), have received considerable attention over the past five decades in various fields, ranging from electronics to medicine, due to their unique physicochemical, electronic, optical, and mechanical properties [40,41,42]. Mostly, polymers are employed as a matrix for physical adsorption or covalent immobilization of enzymes [40,41,42]. It has been shown that a positively charged self-assembled monolayer (SAM) of cysteamine formed on AuNPs immobilized on the electrode coated with PANI is a favorable matrix for the immobilization of dehydrogenase and enhancement of electron transfer [24]. Conducting polymers can enhance charge transfer by facilitating improved electrical contact between GOx(FAD) and the working electrode [2]. Additionally, such polymers contribute to enhancing the selectivity of biosensors in the presence of various electrochemically active substances [39,41]. Conducting polymers can be formed by chemical [41], electrochemical [35,39,42], and enzyme-mediated [43,44,45] synthesis. Polymers formed through enzyme-mediated synthesis often exhibit a wide molecular weight distribution [45]. Polyaniline is characterized by three oxidation stages: leucomeraldine (fully reduced form), emeraldine (semi-reduced and semi-oxidized forms), and pernigraniline (fully oxidized form). Due to its electrocatalytic activity, polyaniline is a very promising matrix for biosensing [40].

Various hybrid composites have been successfully applied in the development of biosensors [35,36,37]. PANI composites with embedded gold nanorods [34] or montmorillonite and PtNPs [35] are considered an excellent matrix for GOx immobilization, primarily because of their large specific surface area and high electroactivity. The application of gold nanomaterials, both with [16,32] and without Ppy [30], as well as polymeric nanocomposites [43,44] in the construction of electrochemical glucose biosensors has been presented previously. The developed mediator-free glucose biosensor based on a glassy carbon (GC) electrode modified by NPAu and GOx (GC/NPAu/GOx) exhibited a sensitivity of 12.1 μA/(mM cm^2^) [13]. The sensitive (32.52 μA/(mM cm^2^)) enzymatic glucose biosensor was obtained using a GC electrode modified by AuNPs and ionic liquids-based polysome (Au@ILs-polysome) nanocomposites (GC/Au@ILs-polysome/GOx) [25]. The DET from GOx to gold (Au) disk electrode modified by Fe@Au-cysteamine (Au/Fe@Au-cysteamine/GOx) was investigated, and its sensitivity was 0.057 μA/mM [28].

Glucose biosensors are undoubtedly among the most popular sensors on the market. This study introduces a novel enzyme immobilization matrix designed to integrate PANI nanocomposites with embedded AuNPs and GOx (PANI-AuNPs-GOx), along with DGNS and cystamine (Cys) SAM, aimed to develop mediator-free enzymatic glucose biosensors. The performance of the developed biosensors and their analytic characteristics, excellent stability, resistance to interfering compounds, and successful applicability for glucose determination in the serum were determined.

## 2. Materials and Methods

### 2.1. Materials

Glucose oxidase (type VII, from *Aspergillus niger*, 208 units/mg protein) and cystamine dihydrochloride were purchased from Fluka (Buchs, Switzerland), D-(+)-glucose, D(+)-saccharose, D(+)-xylose, D(+)-galactose, D(+)-mannose, D(-)-fructose, tannic acid, and potassium hexacyanoferrate(II) trihydrate (K_4_[Fe(CN)_6_]·3H_2_O)—from Carl Roth GmbH+Co.KG (Karlsruhe, Germany). Tetrachloroauric acid trihydrate (HAuCl_4_·3 H_2_O) and sodium citrate were obtained from Alfa Aesar GmbH&Co KG (Karlsruhe, Germany) and Penta (Praha, Czech Republic), respectively. The solution of 0.05 M sodium acetate (SA) buffer was prepared from sodium acetate trihydrate (CH_3_COONa·3H_2_O, from Reanal (Budapest, Hungary)) and potassium chloride (KCl, from Lachema (Neratovice, Czech Republic)). Aniline, sodium hydroxide, and potassium hydroxide were sourced from Merck KGaA (Darmstadt, Germany) and Reanal (Budapest, Hungary), respectively. Potassium nitrate (KNO_3_) and potassium hexacyanoferrate(III) (K_3_[Fe(CN)_6_]) were purchased from Acros Organics (Morris Plains, NJ, USA). A graphite rod (GR, 3 mm in diameter, 0.071 cm^2^ area), H_2_O_2,_ and hydrochloric acid were obtained from Sigma-Aldrich (Saint Louis, MO, USA). GR electrodes were polished with powder of α-aluminium oxide (Al_2_O_3_, 0.3 μm, type N) purchased from Electron Microscopy Sciences (Hatfield, MA, USA). A 25% solution of glutaraldehyde (GA) was received from Fluka Chemie GmbH (Buchs, Switzerland), while L-ascorbic acid (AA) and uric acid (UA) were obtained from AppliChem GmbH (Darmstadt, Germany). All chemicals used in the investigations were of either analytical grade or the highest quality. Solutions of sugars were prepared one day before measurements. Aniline was subjected to purification using a 5 cm column filled with Al_2_O_3_ powder.

### 2.2. Synthesis of AuNPs and PANI-AuNPs-GOx Nanocomposites

First of all, the synthesis of 6 nm AuNPs (2.3 × 10^16^ particles/L) was carried out following the procedure outlined in our previous study [16], with detailed information available in Supplementary Materials. A two-day enzyme-assisted synthesis of PANI-AuNPs-GOx nanocomposites was performed based on the methodology described in [44] and detailed in Supplementary Materials. Formed polymer nanocomposites were dispersed in 40 μL of SA buffer in an ultrasonic bath from Bandelin Electronic GmbH & Co. KG (Berlin, Germany) and used for study.

### 2.3. The Pre-Treatment and Modification of GR Electrode

**Figure 1:**
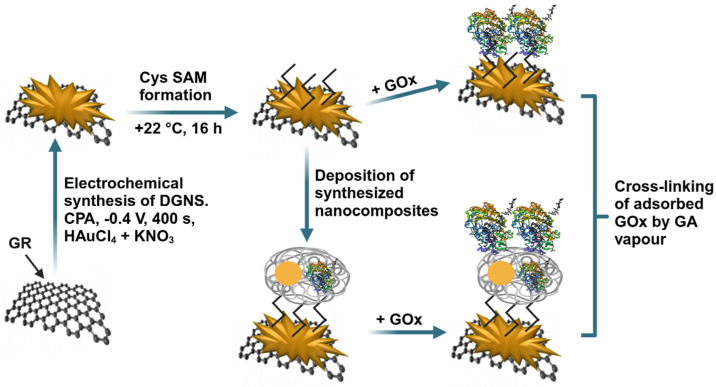
Schematic representation of glucose biosensors based on GR/DGNS/Cys/GOx and GR/DGNS/Cys/PANI-AuNPs-GOx/GOx electrodes.

The graphite rod was polished with fine emery paper and Al_2_O_3_ powder. After rinsing, it was sealed in a silicone tube. GR electrode was then modified with DGNS, which were synthesized using a computerized potentiostat/galvanostat (Autolab/PGSTAT 302 N, EcoChemie, Utrecht, The Netherlands) with GPES 4.9 software (AUT83239). The DGNS were synthesized from a stirred (1200 rpm) 0.1 M KNO_3_ solution consisting of 6 mM HAuCl_4_ by applying a constant potential of +0.4 V for 400 s [30]. Further, a Cys SAM was formed on the surface of the GR electrode modified with DGNS (GR/DGNS). Next, 3 µL of 25 mg/mL glucose oxidase was deposited on the surface, followed by water evaporation and cross-linking of the GOx and Cys amine groups. For this, the electrode was exposed to 25% GA solution vapor for 15 min at room temperature to improve enzyme immobilization. The resulting GR/DGNS/Cys/GOx electrode was thoroughly washed with distilled water to remove any unbound GOx molecules. For the preparation of the GR/DGNS/Cys/PANI-AuNPs-GOx/GOx electrode, 3 µL of PANI-AuNPs-GOx nanocomposites were deposited onto the GR/DGNS/Cys electrode, followed by GOx deposition and cross-linking with GA, as previously described. A schematic representation of the preparation process for both GR/DGNS/Cys/GOx and GR/DGNS/Cys/PANI-AuNPs-GOx/GOx electrodes is shown in Figure 1.

### 2.4. Investigation of Electrochemical Characteristics of Glucose Biosensors

Constant potential amperometry (CPA) and cyclic voltammetry (CV) measurements were conducted in a 0.05 M SA buffer, pH 5.6, containing 0.1 M KCl, using a potentiostat/galvanostat. All measurements were performed using a three-electrode system, consisting of a modified GR electrode as the working electrode, a 2 cm^2^ platinum (Pt) wire (BASi Research Products, West Lafayette, IN, USA) as the auxiliary electrode, and an Ag/AgCl_(3 M KCl)_ electrode as the reference.

The optimal conditions were selected using the glucose biosensor based on the GR/DGNS/Cys/GOx electrode. The concentration of the Cys solution, the temperature, the time of the GR/DGNS electrode incubation, and the applied potential affect the sensitivity of the biosensor. The GR/DGNS electrodes were kept for 25 h at +22 °C in Cys solutions of different concentrations, from 1.0 to 50 mM, to determine the optimal Cys concentration. The GR/DGNS electrodes were stored in a 5.0 mM Cys solution from 4 to 48 h at +22 °C to choose the optimal incubation time. The optimal temperature was tested in the range of +4 to +30 °C with incubation for 16 h. The current responses to glucose in these experiments were registered by CPA at −0.30 V. The optimal applied potential was selected using CPA at potentials ranging from −0.70 to −0.20 V.

All CV measurements were performed in an unstirred 0.05 M SA buffer with 0.1 M KCl in the potential range from −0.60 to +0.60 V, the step potential being 0.0024 V, and the potential scan rate 0.05 V/s. The electrocatalytic activity towards H_2_O_2_ was evaluated using the GR/DGNS/Cys, GR/DGNS/Cys/GOx, and GR/DGNS/Cys/PANI-AuNPs-GOx/GOx electrodes. The cyclic voltammograms of glucose biosensors in the presence and absence of O_2_ (the solution was deaerated by passing argon for 60 min) were registered using bare GR, GR/DGNS, GR/DGNS/Cys, GR/DGNS/Cys/GOx, and GR/DGNS/Cys/PANI-AuNPs-GOx/GOx electrodes.

All results of the CPA measurements were repeated at least three times and evaluated as an average value with error bars. The intercept, slope, determination coefficient (*R*^2^) of the calibration curve, maximal current (Δ*I*_max_), and apparent Michaelis constant (*K*_M(app)_) were estimated using SigmaPlot software 12.5. The results obtained were approximated using the hyperbolic function (*y* = *ax*/(*b* + *x*))*,* where the parameters *a* and *b* were Δ*I*_max_ and *K*_M(app)_, respectively. Sensitivity was calculated from the slope of the linear plot relative to the square of the GR area.

### 2.5. The Evaluation of the Surface Area of Modified Electrodes

The electroactive surface area (EASA) of the electrodes was evaluated using CV in a potential range from −0.80 to +0.80 V and various potential scan rates (0.010, 0.025, 0.050, 0.075, 0.100, 0.125, 0.150, and 0.175 V/s) in the solution of 2.5 mM K_3_[Fe(CN)_6_] and K_4_[Fe(CN)_6_] with 0.1 M KCl. The EASA was calculated according to the Randles–Sevcik equation [46]:

*I*_p_ = 2.69 × 10^5^∙n^3/2^∙EASA∙*D*^1/2^∙*C*∙*v*^1/2^ (1)

where *I*_p_—the maximal peak current (A), n—the number of electrons appearing in the half-reaction for the redox pair ([Fe(CN)_6_]^3−^ + e^−^ ⇆ [Fe(CN)_6_]^4−^), *D*—the diffusion coefficient (7.63 × 10^−6^ cm^2^/s [47]), *C*—the concentration of electroactive species (0.0000025 mol/cm^3^), and *v*—the potential scan rate (V/s).

### 2.6. The Stability and Practical Application of Glucose Biosensors

To evaluate the stability of glucose biosensors, GR/DGNS/Cys/GOx and GR/DGNS/Cys/PANI-AuNPs-GOx/GOx electrodes were stored over an SA buffer solution (pH 5.6) at +4 °C for 1, 5, 8, 12, 19, 26, 40, 47, 55, and 71 days before measurements. After each mentioned period, the GR/DGNS/Cys/GOx and GR/DGNS/Cys/PANI-AuNPs-GOx/GOx electrodes were used to evaluate the changes in the current response to glucose using the CPA method at −0.35 V vs. Ag/AgCl_(3 M KCl)_. After that, the working electrodes were washed with distilled water, dried at room temperature, and stored over an SA buffer solution (pH 5.6) at +4 °C until the next experiment. The selectivity and impact of electrochemically active interfering compounds on the GR/DGNS/Cys/GOx and GR/DGNS/Cys/PANI-AuNPs-GOx/GOx electrodes were evaluated. The quantitative determination of glucose in diluted blood serum using the GR/DGNS/Cys/PANI-AuNPs-GOx/GOx electrode was performed by the CPA method at −0.35 V vs. Ag/AgCl_(3 M KCl)_, as described earlier [43,44]. The blood serum sample was diluted by SA buffer at a ratio of 1:10 and centrifuged using an IEC CL31R Multispeed centrifuge from Thermo Electron Industries S.A.S. (Château-Gontier-sur-Mayenne, France) for 8 min (14.6 × 10^3^× *g*). The influence of various sugars on the current response was investigated in blood serum with 0.5 and 2.0 mM of glucose before and after the addition of 1.0 mM of fructose, mannose, xylose, saccharose, and galactose. The serum with 3.0 mM of glucose, with 3.0 mM of glucose and 0.01, 0.05, 0.1, or 0.2 mM of AA, or with 3 mM of glucose and 0.01 or 0.025 mM of UA was used to assess the effects of ascorbic and uric acids on the current response in the presence of glucose. Glucose determination in a 10-fold diluted sample of blood serum was conducted under optimal conditions using the GR/DGNS/Cys/PANI-AuNPs-GOx/GOx electrode. The addition method was employed to accurately calculate the concentration of glucose.

## 3. Results and Discussion

### 3.1. The Optimization of Biosensor Performance

The selection of optimal conditions of Cys SAM formation on the surface of DGNS to improve the performance of the mediator-free glucose biosensor was carried out using the GR/DGNS/Cys/GOx electrode according to procedures described in Section 2.4.

**Figure 2:**
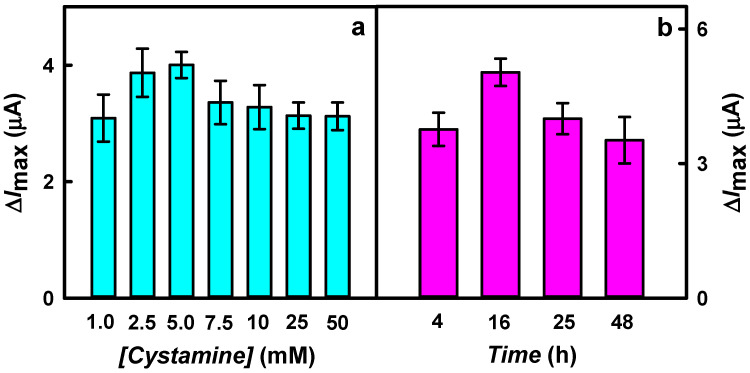
Effect of cystamine concentration (cyan color) (**a**) and the incubation time (violet color) (**b**) of GR/DGNS electrode on the current response to glucose. Conditions: (**a**) 25 h of incubation in Cys solutions of different concentrations; (**b**) an incubation in 5.0 mM Cys solution at +22 °C. CPA-based current responses were registered using GR/DGNS/Cys/GOx electrode in 0.05 M SA buffer with 0.1 M KCl at −0.30 V vs. Ag/AgCl_(3 M KCl)_.

Firstly, the influence of Cys concentration on the reduction current response of the developed glucose biosensor was evaluated. As shown in Figure 2a, the highest current response to glucose was achieved after the incubation of the modified electrode in 5.0 mM Cys solution (4.00 ± 0.22 μA). In addition, the highest value of Δ*I*_max_ was about 1.3 times higher compared to the results obtained for electrodes for which a SAM was formed using 1.0 and 50 mM Cys solutions (3.09 ± 0.63 and 3.12 ± 0.24 μA), respectively.

In the second step, the optimal incubation time in the Cys solution was selected. Incubating the electrodes from 4 to 16 h increased the Δ*I*_max_ by 1.34 times (from 3.76 ± 0.37 to 5.04 ± 0.31 μA) (Figure 2b). Meanwhile, a subsequent increase of incubation time up to 48 h resulted in a decrease of current response by 1.43 times (3.52 ± 0.52 μA).

**Figure 3:**
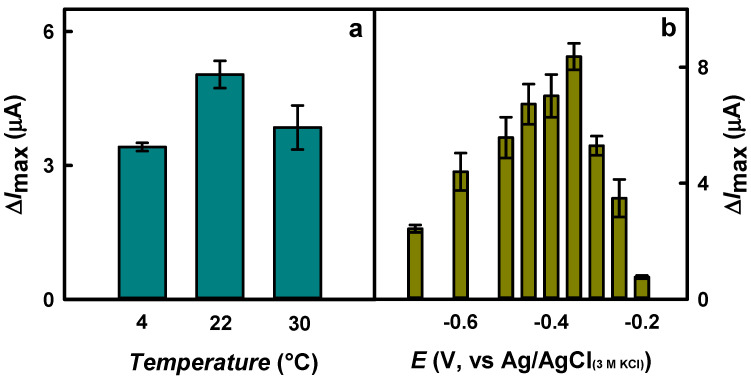
The influence of 5.0 mM Cys solution temperature (emerald color) (**a**) and applied potential (mustard color) (**b**) on the current response to glucose. Conditions: (**a**) 16 h of an incubation, −0.30 V vs. Ag/AgCl_(3 M KCl)_; (**b**) an incubation at 22 °C. CPA-based current responses were registered using GR/DGNS/Cys/GOx electrode in 0.05 M SA buffer with 0.1 M KCl.

To evaluate the optimal incubation temperature, SAM was formed using 5.0 mM Cys solution at +4, +22, and +30 °C. As shown in Figure 3a, the Δ*I*_max_ to glucose after incubation at +22 °C (5.04 ± 0.31 μA) was 1.48 and 1.31 times higher than that obtained by incubating the electrode at 0 °C (3.41 ± 0.09 μA) or +30 °C (3.85 ± 0.49 μA), respectively.

Finally, the influence of the applied potential on current responses to glucose was investigated. Figure 3b illustrates that the Δ*I*_max_ at −0.35 V (8.37 ± 0.46 μA) was 3.44 and 11.0 times higher compared to the current responses registered at −0.70 V (2.43 ± 0.13 μA) and −0.20 V (0.764 ± 0.056 μA), respectively. A low value of the applied potential is suitable for reducing the effect of electroactive compounds present in the serum or other samples. The groups of scientists declared an applied potential of −0.27 V vs. Ag/AgCl_(3 M KCl)_ for glucose biosensing using an Au/Fe@Au-cysteamine/GOx electrode [28] and −0.45 V vs. Ag/AgCl_(sat.)_ using a magnetic screen-printed electrode modified by RGO-Fe_3_O_4_/GOx [11]. Glucose detection was performed using a gold chip modified with copper (Cu) nanoflower (Cu-nanoflower), cysteamine-AuNPs, polyvinyl alcohol (PVA), graphene oxide (GO) nanofibers (NFs), and immobilized GOx and horseradish peroxidase (HRP) (Au/PVA-GO NFs/cysteamine-AuNPs/Cu-nanoflower/GOx-HRP) at an applied potential of −0.25 V vs. Ag/AgCl [14]. An applied potential of −0.5 V vs. Ag/AgCl_(3 M KCl)_ was used for the glucose biosensor based on a gold electrode modified by gold nanopine needles (AuNNs), β-cysteamine, and the mixture of GOx, bull serum albumin (BSA), and poly(ethylene glycol) diglycidylether (PEGDE) (Au/AuNNs/cysteamine/GOx-BSA-PEGDE) [31]. A low value of applied potential is suitable for decreasing the inference of electroactive compounds, whose effect increases at the positive potential [36,41]. For further investigations, Cys SAM formation was performed using 5 mM Cys solution for 16 h at +22 °C. Moreover, an applied potential of −0.35 V vs. Ag/AgCl_(3 M KCl)_ was selected to register the current response to glucose.

Fabricated GR/DGNS/Cys, GR/DGNS/Cys/GOx, and GR/DGNS/Cys/PANI-AuNPs-GOx/GOx electrodes were examined for electrocatalytic activity towards H_2_O_2_ (Figures S1 and S2). The highest response to H_2_O_2_ was observed using the GR/DGNS/Cys electrode. Moreover, the anodic and cathodic peaks appeared at +0.032 and −0.25 V vs. Ag/AgCl_(3 M KCl)_, respectively. The oxidation and reduction peaks were monitored at +0.40 and −0.27 V vs. Ag/AgCl_(3 M KCl)_ for the GR/DGNS/Cys/GOx electrode and at +0.22 and −0.26 V vs. Ag/AgCl_(3 M KCl)_ for the GR/DGNS/Cys/PANI-AuNPs-GOx/GOx electrode. Figure S2b presents the current responses to H_2_O_2_ obtained for the GR/DGNS/Cys, GR/DGNS/Cys/GOx, and GR/DGNS/Cys/PANI-AuNPs-GOx/GOx electrodes using CPA at −0.35 V vs. Ag/AgCl_(3 M KCl)_. As can be seen from the results presented, the GR/DGNS/Cys electrode was characterized by 7.46 and 12.0 times better electrocatalytic activity towards 1.0 mM H_2_O_2_ than the GR/DGNS/Cys/PANI-AuNPs-GOx/GOx and GR/DGNS/Cys/GOx electrodes, respectively.

In addition, the GR, GR/DGNs, GR/DGNS/Cys, GR/DGNS/Cys/GOx, and GR/DGNS/Cys/PANI-AuNPs-GOx electrodes were characterized by a reduction of O_2_ (Figure S3). In the absence of O_2_ (Figure S3b), the reduction peaks, which were observed in the presence of O_2_ (Figure S3a), practically disappeared for all tested electrodes. Considering the electrocatalytic activity of fabricated electrodes towards H_2_O_2_ and O_2_, it can be summarized that in the case of fabricated enzymatic electrodes, the recorded current response during glucose biosensing is the sum of the signals generated during the reduction of H_2_O_2_ and O_2_. The increase in current at a negative applied potential due to the reduction of H_2_O_2_ is fully compensated by a decrease in O_2_ concentration due to O_2_ consumption during the enzymatic reaction.

### 3.2. The Comparison and Characterization of Glucose Biosensors Based on Differently Modified Electrodes

The EASA of the GR/DGNS/Cys/GOx and GR/DGNS/Cys/PANI-AuNPs-GOx/GOx electrodes was investigated according to the procedures described in Section 2.5.

**Figure 4:**
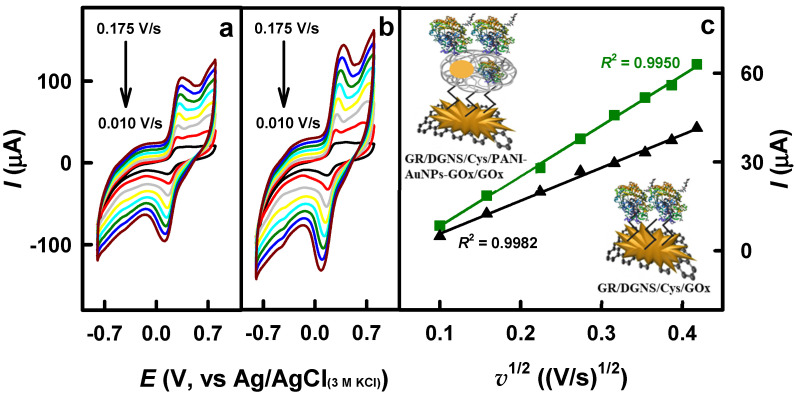
Cyclic voltammograms of GR/DGNS/Cys/GOx (**a**) and GR/DGNS/Cys/PANI-AuNPs-GOx/GOx (**b**) electrodes were recorded at potential scan rates ranging from 0.010 to 0.175 V/s (from black to brown colors), and the relationship between the square root of the potential scan rate and peak anodic current was analyzed (**c**). Conditions: (**c**) GR/DGNS/Cys/PANI-AuNPs-GOx/GOx (green line) and GR/DGNS/Cys/GOx (black line) electrodes. The measurements were performed in 2.5 mM K_3_[Fe(CN)_6_] and K_4_[Fe(CN)_6_] solution containing 0.1 M KCl.

The recorded cyclic voltammograms (Figure 4a,b) were characterized by reversible anodic and cathodic peaks, as well as an increase in the magnitude of peak separation with increasing potential scan rate. The EASA of the GR/DGNS/Cys/GOx and GR/DGNS/Cys/PANI-AuNPs-GOx/GOx electrodes was calculated using the slope of the lines (Figure 4c) and was 0.060 and 0.092 cm^2^, respectively. It is seen that PANI-AuNPs-GOx nanocomposites increase the electroactive surface area of the developed electrode by 1.53 times, ensuring better spatial orientation of GOx on the surface and making it an excellent matrix for GOx immobilization.

**Figure 5:**
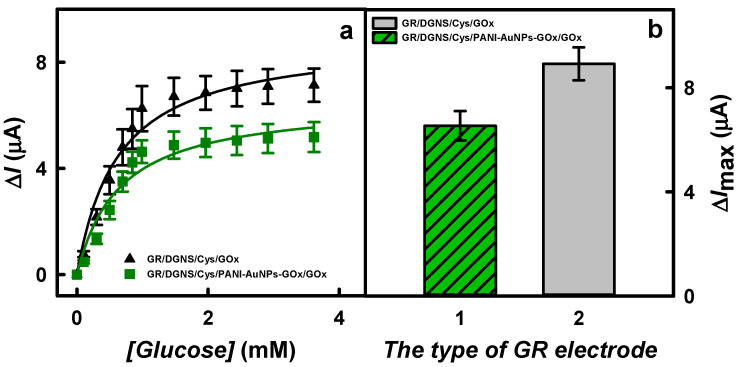
The calibration plots (**a**) and diagrams of the calculated maximal current responses (**b**) of glucose biosensors based on differently modified electrodes. Conditions: (**b**) GR/DGNS/Cys/PANI-AuNPs-GOx/GOx (1 column) and GR/DGNS/Cys/GOx (2 column) electrodes. CPA was used to register the current response in 0.05 M SA buffer with 0.1 M KCl at −0.35 V vs. Ag/AgCl_(3 M KCl)_.

Furthermore, the biosensors’ current responses to the glucose concentrations up to 3.62 mM were investigated and are presented in Figure 5a. It was observed that all the dependencies followed a hyperbolic function and agreed with Michaelis–Menten kinetics. The observed decrease in the registered current at −0.35 V vs. Ag/AgCl_(3 M KCl)_ with increasing glucose concentration was attributed to O_2_ consumption during the enzymatic oxidation of glucose by GOx. The decrease in the registered current at −0.35 V vs. Ag/AgCl_(3 M KCl)_ with increasing concentration of glucose is due to the consumption of O_2_ during the enzymatic oxidation of glucose by GOx [21,23]. The decrease in O_2_ concentration during H_2_O_2_ production (O_2_ + 2H^+^ + 2e^−^ → H_2_O_2_) is due to the electrochemical reduction of O_2_ (O_2_ + 4H^+^ + 4e^−^ → 2H_2_O), which has a much stronger effect on the registered current than the reduction of H_2_O_2_ formed during the enzymatic reaction (H_2_O_2_ + 2e^−^ + 2H^+^ → 2H_2_O), which is based on the transfer of a lower number of electrons. In addition, particularly at negative potentials, the thermodynamic reduction of H_2_O_2_ is less favorable than the reduction of O_2_ under the same conditions [12,23]. The values of *K*_M(app)_ calculated for the GR/DGNS/Cys/GOx and GR/DGNS/Cys/PANI-AuNPs-GOx/GOx electrodes were 0.539 and 0.575 mM, respectively. The lower values of *K*_M(app)_ could indicate the higher catalytic activity and higher affinity of GOx [34].

The Δ*I*_max_’s characterized for the GR/DGNS/Cys/GOx and GR/DGNS/Cys/PANI-AuNPs-GOx/GOx electrodes are presented in Figure 5b. The Δ*I*_max_ of the biosensor based on the GR/DGNS/Cys/GOx electrode (8.92 ± 0.77 μA) was 1.37 times higher than that obtained using the GR/DGNS/Cys/PANI-AuNPs-GOx/GOx (6.53 ± 0.71 μA) electrode. The lower current responses observed using the glucose biosensor based on the GR/DGNS/Cys/PANI-AuNPs-GOx/GOx electrode can be explained by less efficient electron transfer between GOx and the working electrode through the layer of polymeric nanocomposites.

**Figure 6:**
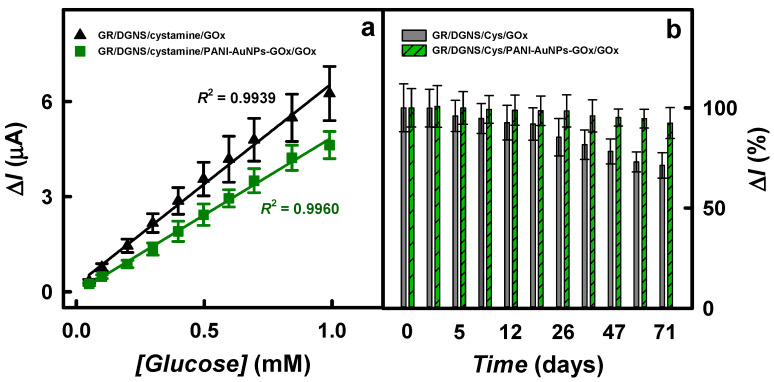
The linear range (**a**) and the diagrams of the difference in current responses to 2.91 mM of glucose over time (**b**) for biosensors based on GR/DGNS/Cys/GOx and GR/DGNS/Cys/PANI-AuNPs-GOx/GOx electrodes. CPA was used to register the current response in 0.05 M SA buffer with 0.1 M KCl at −0.35 V vs. Ag/AgCl_(3 M KCl)_.

The analytical characteristics, including the linear range (LR) and *R*^2^, the sensitivity, and the limit of detection (LOD) of the glucose biosensors based on GR/DGNS/Cys/GOx or GR/DGNS/Cys/PANI-AuNPs-GOx/GOx electrodes were evaluated and compared (Figure 6a and Table 1).

As evident, the LR for both electrodes was up to 1.0 mM. The *R*^2^ for the GR/DGNS/Cys/GOx and GR/DGNS/Cys/PANI-AuNPs-GOx/GOx electrodes was 0.9939 and 0.9960, respectively. The linear range for the developed biosensors was 2.0, 3.13, 3.57, 4.0, and 10.0 times wider (Table 1) than that obtained for the GC/Au@ILs-polysome/GOx electrode (up to 0.5 mM) [25], for the gold electrode modified by AuNC-embedded dual-enzyme (GOx and HRP) nanoparticles (Au/AuNC-DENPs, up to 0.32 mM) [18], for the carbon paste (CP) electrode modified by AuNPs_(24 nm)_ and GOx (CP/AuNPs_(24 nm)_/GOx, up to 0.28 mM) [19], for the Au/AuNNs/cysteamine/GOx-BSA-PEGDE electrode (up to 0.25 mM) [31], and for the Au/PVA-GO NFs/cysteamine-AuNPs/Cu-nanoflower/GOx-HRP electrode (up to 0.10 mM) [14], respectively. The LR evaluated here for the developed biosensors based on the GR/DGNS/Cys/PANI-AuNPs-GOx/GOx electrode was the same as for the Pt electrode modified by PANI and gold nanorod composite and GOx (Pt/PANI/gold nanorod/GOx) [34].

The developed glucose biosensors based on GR/DGNS/Cys/GOx and GR/DGNS/Cys/PANI-AuNPs-GOx/GOx electrodes were characterized by high sensitivity: 93.7 and 72.0 μA/(mM cm^2^**)** (Table 1). The lower sensitivity of the GR/DGNS/Cys/PANI-AuNPs-GOx/GOx electrode can be explained by the presence of a polymeric layer, which interferes with electron transfer [41]. Glucose biosensors based on GR/DGNS/Cys/GOx and GR/DGNS/Cys/PANI-AuNPs-GOx/GOx electrodes were 7.74 and 5.95 times more sensitive than glucose biosensor based on GC/NPAu/GOx electrode (12.1 μA/(mM cm^2^)) [13]. The sensitivity of the glucose biosensor based on the GR/DGNS/Cys/PANI-AuNPs-GOx/GOx electrode (72.0 μA/(mM cm^2^) or 5.08 μA/mM) was 2.21 times higher than that of the GC/Au@ILs-polysome/GOx electrode (32.52 μA/(mM cm^2^)) [25], 5.22 times higher than that of the Pt/PANI/gold nanorod/GOx electrode (13.8 μA/(mM cm^2^)) [34], and 23.4 times higher than that of the GC electrode modified with a film of overoxidized polypyrrole (OOPpy), decorated with AuNPs and immobilized GOx (GC/OOPpy-AuNPs/GOx, 0.217 μA/mM) [21]. The electrodes developed in this study were more sensitive than the Au/AuNC-DENPs electrode (18.944 μA/(mM cm^2^)) [18].

The GR/DGNS/Cys/PANI-AuNPs-GOx/GOx and GR/DGNS/Cys/GOx electrodes demonstrated a relative standard deviation (RSD) of 9.03 and 13.6% in repeated measurements of 1.0 mM glucose. A 95% registered current response to glucose for the developed electrodes was recorded in about 5 s, which is 2.2 times faster than for the GC/OOPpy-AuNPs/GOx (11 s) electrode [21]. The value of LOD was estimated as the lowest glucose concentration, at which the current response exceeds the background value plus 3 σ. The glucose biosensor based on the GR/DGNS/Cys/GOx electrode was characterized by a 1.26 times lower LOD than that calculated for the biosensor based on the GR/DGNS/Cys/PANI-AuNPs-GOx/GOx electrode. The LOD values obtained for developed glucose biosensors based on GR/DGNS/Cys/GOx and GR/DGNS/Cys/PANI-AuNPs-GOx/GOx electrodes were 6.67 and 5.29 times lower compared to the LOD declared for glucose biosensor based on gold electrode modified with graphene/AuNPs/chitosan composites and immobilized GOx (0.18 mM) [23]. The GR/DGNS/Cys/PANI-AuNPs-GOx/GOx electrode developed here was characterized by a 14.7 times lower value of LOD compared to the results obtained for the GC/OOPpy-AuNPs/GOx electrode (LOD was 0.5 mM) [21].

Effective immobilization preserves enzyme activity and increases the stability of biosensors. The stability of glucose biosensors was examined by monitoring current responses according to the procedures described in Section 2.6. Figure 6b presents the current response over time for the developed electrodes. The current responses of the GR/DGNS/Cys/GOx and GR/DGNS/Cys/PANI-AuNPs-GOx/GOx electrodes after 71 days decreased by 28.7 and 7.62%, respectively, which is better than that of the Au/AuNC-DENPs electrode (30% of current responses decay after 26 days) [18]. The higher stability of the GR/DGNS/Cys/PANI-AuNPs-GOx/GOx electrode might be attributed to the biocompatibility of the polymeric layer, which created a suitable environment for the immobilized enzyme [4,21]. The developed glucose biosensor based on the GR/DGNS/Cys/PANI-AuNPs-GOx/GOx electrode was more stable than glucose biosensors based on GC/Au@ILs-polysome/GOx (10 days) [25], GC/OOPpy-AuNPs/GOx (more than 14 days) [21], Au/PVA-GO NFs/cysteamine-AuNPs/Cu-nanoflower/GOx-HRP (91.0% of the initial activity reached after 20 days) [14], and on Au/AuNNs/cysteamine/GOx-BSA-PEGDE (30 days) [31] electrodes. The mediator-free glucose biosensor based on the GR/DGNS/Cys/PANI-AuNPs-GOx/GOx electrode was more stable than the GR electrode modified by PANI-AuNPs-GOx (2 days) [44] or GR/PANI-AuNPs-GOx/GOx (22 days) [43] in the presence of the redox mediator phenazine methosulfate (PMS).

Glucose biosensors based on GR/DGNS/Cys/GOx and GR/DGNS/Cys/PANI-AuNPs-GOx/GOx electrodes were characterized by good repeatability of 8.96 and 8.01% for 8 measurements (Figure S4). The registered current responses to 0.50 mM glucose after 8 measurements were changed no more than 1.16 times compared with the results of the first measurement. The GR/DGNS/Cys/PANI-AuNPs-GOx/GOx electrode, with its high stability and good repeatability, is more attractive for practical applications.

### 3.3. Determination of Glucose in a Serum Sample

Interfering species could oxidase during electrochemical glucose sensing, resulting in impaired detection of the analyte and affecting the biosensor current response [31]. However, polymer–nanoparticle nanocomposites reduce the impact of various electrochemically active interfering substances due to the polymer’s presence [39,41] and facilitate charge transfer due to AuNPs incorporated in the polymer [7]. To evaluate the selectivity of biosensors based on GR/DGNS/Cys/GOx and GR/DGNS/Cys/PANI-AuNPs-GOx/GOx electrodes, the effect of various sugars on the current response to glucose was investigated according to the procedures presented in Section 2.6. As is seen from Figure S5a in the case of the GR/DGNS/Cys/GOx electrode, the addition of 1.0 mM of fructose, mannose, xylose, saccharose, or galactose had no significant or no effect on the registered signal. No obvious effect of various sugars was observed for the GR/DGNS/Cys/PANI-AuNPs-GOx/GOx electrode (Figure S5b).

**Figure 7:**
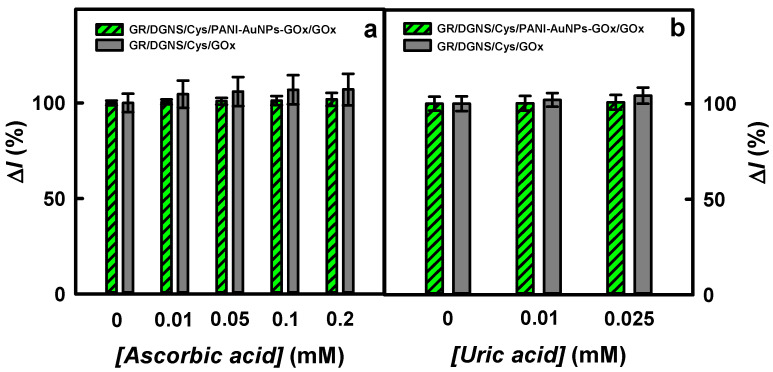
The influence of ascorbic (**a**) and uric (**b**) acids on the current response of glucose biosensor based on GR/DGNS/Cys/GOx and GR/DGNS/Cys/PANI-AuNPs-GOx/GOx electrodes. Conditions: 10-fold diluted samples of blood serum containing 3.0 mM of glucose without and with 0.01, 0.05, 0.10, or 0.20 mM of AA (**a**), or containing 3.0 mM glucose with 0.01 or 0.025 mM of UA (**b**).

It has been declared that ascorbic and uric acids have an impact on the correct detection of glucose in a sample [11,48]. The physiological blood glucose concentration in a nondiabetic person is less than 6.0 mM [21], and up to 30 mM [1] in a diabetic patient, which is much higher than possible concentrations of ascorbic (0.141 mM [49]) and uric (0.1 mM [36]) acids. The current responses to AA or UA were normalized to the current response (100%) to glucose. No significant effect of AA on the current responses of the biosensor based on the GR/DGNS/Cys/PANI-AuNPs-GOx/GOx electrode was observed (Figure 7a). The addition of 0.20 mM AA (14.2 times higher concentration than in 10 times diluted serum) to a diluted sample of blood serum containing 3.0 mM of glucose increased the current response by 1.82% compared to the result observed for the matrix without AA. The impact of 0.20 mM AA on the registered signal was about 2.80 times less using the GR/DGNS/Cys/PANI-AuNPs-GOx/GOx electrode than the CP/AuNPs_(24 nm)_/GOx electrode (an interference of 5.1% was reported for 0.36 mM of AA) [19]. The developed biosensor was 2.60, 2.75, and 3.38 times more resistant to 0.20 mM AA than biosensors based on the GR/PANI-AuNPs-GOx/GOx electrode in the presence of PMS (the interference of 4.74% was monitored for 0.20 mM AA) [43]; the GR electrode modified with AuNPs_(3.5 nm)_, GOx, and Ppy in the presence of redox mediator-1,10-phentroline-5,6-dione (PD) (GR/AuNPs_(3.5 nm)_/PD/GOx/Ppy, 5.00% interference was monitored for 0.05 mM of AA) [16]; and the GR electrode modified with Ppy nanocomposites based on AuNPs and GOx (GR/Ppy-AuNPs_(AuCl4_-_)_-GOx) in the presence of PMS (6.16% interference was monitored for 0.10 mM AA) [44]. The addition of 0.01, 0.05, 0.1, or 0.2 mM of AA in the diluted samples of blood serum with 3.0 mM of glucose increased the current responses registered using the GR/DGNS/Cys/GOx electrode by 4.58, 5.95, 6.88, or 6.99% compared to the responses without AA.

Uric acid at the selected concentrations also did not affect the current responses of the glucose biosensor based on the GR/DGNS/Cys/PANI-AuNPs-GOx/GOx electrode and had no significant effect on the GR/DGNS/Cys/GOx electrode (Figure 7b). After adding 3.0 mM of glucose with 0.01 or 0.025 mM of UA, the current responses increased by 0.18 and 0.73% for the GR/DGNS/Cys/PANI-AuNPs-GOx/GOx electrode, and 1.98 and 4.22% for the GR/DGNS/Cys/GOx electrode, compared to the results obtained after adding 3.0 mM of glucose without UA. Usually, an interference of less than 10% for electroactive species is considered acceptable [1]. The impact of 0.025 mM of UA (2.5 times higher concentration than it is in 10-fold diluted serum) on the current response of the mediator-free glucose biosensor based on the GR/DGNS/Cys/PANI-AuNPs-GOx/GOx electrode was less than that declared for the GR/AuNPs_(3.5 nm)_/PD/GOx/Ppy electrode (an interference of 9.00% was monitored for 0.1 mM UA) [16] or for the GR/Ppy-AuNPs_(AuCl4_-_)_-GOx electrode in the presence of PMS (an interference of 13.4% was monitored for 0.05 mM AA) [44]. PANI-AuNPs-GOx nanocomposites exhibit strong resistance to higher concentrations of AA and UA than typically found in 10-fold diluted blood serum during glucose biosensing, consistent with the stability characteristics of PANI/GOx nanostructures [41]. The developed glucose biosensor based on the GR/DGNS/Cys/PANI-AuNPs-GOx/GOx electrode was employed for the determination of glucose in a diluted sample of blood serum to investigate its applicability for real object analysis. The measurements were performed in a 10-fold diluted blood serum with 0.420 mM of glucose by the addition method. The glucose concentration was evaluated as 0.404 ± 0.024 mM with a 96.2% recovery ratio (Figure S6). The results obtained from at least three measurements are presented as averages in Table 2.

The obtained values of the recovery ratio ranged from 96.1 ± 4.49 to 96.6 ± 6.92%. The estimated recovery ratio of the fabricated biosensor was similar to the results obtained with GC/OOPpy-AuNPs/GOx (96%) [21] or Au/PVA-GO NFs/cysteamine-AuNPs/Cu-nanoflower/GOx-HRP (96.59−105.26%) [14] electrodes, and better than that obtained using the GR/Ppy-AuNPs_(AuCl4_-_)_-GOx electrode in the presence of PMS (93.6−94.8%) [44]. The developed glucose biosensor works similarly to commercial sensors. Evaluation criteria for glucose testing devices vary by country, agency, methodology, and glucose concentration [50]. The Food and Drug Administration detection criterion for glucose ≥ 75 mg/dL (4.17 mM) is 98 ± 15%, and the European Medicines Agency criterion for glucose ≥ 100 mg/dL (5.55 mM) is 95 ± 15% [50].

The developed mediator-free glucose biosensors based on GR/DGNS/Cys/GOx or GR/DGNS/Cys/PANI-AuNPs-GOx/GOx electrodes are quite cheap due to the low amount of chemicals required for the fabrication of working electrodes. These biosensors can be distinguished by several advantages: (i) high sensitivity (93.7 and 72.0 μA/(mM cm^2^)) and low limit of detection (0.027 and 0.034 mM); (ii) good reproducibility (13.6 and 9.03% of RSD), repeatability (8.96 and 8.01%), and short duration of measurements (5 s); (iii) high storage stability (over than 71 days) and multiple use. The electrochemical mediator-free glucose biosensor based on the GR/DGNS/Cys/PANI-AuNPs-GOx/GOx electrode can be successfully applied for real samples analysis and in clinical practice for the control of diabetes mellitus due to its high sensitivity and resistance to interfering species.

## 4. Conclusions

Glucose biosensors are increasingly popular due to the rising prevalence of diabetes and advancements in biosensor technology. This paper highlights the development of mediator-free enzymatic glucose biosensors based on GR electrodes modified with electrochemically synthesized DGNS, coated with Cys SAM, and additionally modified with PANI-AuNPs-GOx nanocomposites. Biosensors based on GR/DGNS/Cys/GOx and GR/DGNS/Cys/PANI-AuNPs-GOx/GOx electrodes are characterized by high sensitivity, low LOD, and appropriate storage stability. However, PANI-AuNPs-GOx nanocomposites ensure much higher stability over time. The GR/DGNS/Cys/PANI-AuNPs-GOx/GOx electrode has been demonstrated to be successfully applied for the determination of glucose in serum, even in the presence of interfering species. The technology presented in this paper is expected to pave the way for the development of innovative mediator-free enzymatic glucose biosensors, aimed at clinical assays, general bioanalytical applications, as well as general diagnostic purposes— specifically for the control of glucose concentration in the blood of people with diabetes.

## Figures and Tables

**Table 1:** Comparison of glucose biosensors based on electrodes modified by various nanocomposites.

Working Electrode	LOD (mM)/ Sensitivity (μA/(mM cm^2^))	LR (mM)	Reference
Au/PVA-GO NFs/cysteamine-AuNPs/Cu-nanoflower/GOx-HRP	0.018 × 10^−3^/332.68	0.001–0.10	[14]
GC/NPAu/GOx	0.00102/12.1	0.05–10	[13]
Au/AuNC-DENPs	2.58 ^a^/18.944	5.5–320 ^a^	[18]
Pt/PANI/gold nanorod/GOx	0.0058/13.8	0.0176–1	[34]
Au/AuNNs/cysteamine/GOx-BSA-PEGDE	0.007/–	0.025–0.25	[31]
CP/AuNPs_(24 nm)_/GOx	0.01/8.4 ^b^	0.04–0.28	[19]
GC/Au@ILs-polysome/GOx	0.02/32.52	0.05–0.5	[25]
Au/graphene/AuNPs/chitosan/GOx	0.18/0.55 ^b^	2–10	[23]
GC/OOPpy-AuNPs/GOx	0.5/0.217 ^b^	1.0–8.0	[21]
GR/DGNS/Cys/GOx	0.027/93.7	0.050–1.0	This work
GR/DGNS/Cys/PANI-AuNPs-GOx/GOx	0.034/72.0	0.050–1.0	This work

^a^ The value in nM; ^b^ The sensitivity is in μA/mM. AuNC—gold nanocluster, AuNNs—gold nanopine needles, Au@ILs-polysome—gold nanoparticles and ionic liquids-based polysome nanocomposites, BSA—bull serum albumin, CP—carbon paste, Cu-nanoflower—copper nanoflower, DENPs—dual-enzyme (GOx and HRP) nanoparticles, GC—glassy carbon, GO NFs—graphene oxide nanofibers, HRP—horseradish peroxidase, NPAu—nanoporous gold, OOPpy—overoxidized polypyrrole, PEGDE—poly(ethylene glycol) diglycidylether, and PVA—polyvinyl alcohol.

**Table 2:** Determination of glucose concentration in 10-fold diluted blood serum.

Total Concentration (mM)	Detected * Concentration (mM)	Recovery Ratio (%)
0.466	0.448 ± 0.025	96.1
0.520	0.501 ± 0.027	96.3
0.790	0.763 ± 0.053	96.6

* The responses were registered in 10-fold diluted samples of blood serum at −0.35 V vs. Ag/AgCl_(3 M KCl)_.

## Data Availability

Data are contained within the article.

## Supplementary Materials

The following supporting information can be downloaded at https://www.mdpi.com/article/10.3390/bios15030196/s1, Figure S1: Cyclic voltammetric measurements of GR/DGNS/Cys (a), GR/DGNS/Cys/PANI-AuNPs-GOx/GOx (b), and GR/DGNS/Cys/GOx (c) electrodes in 0.05 M SA buffer with 0.1 M KCl containing various concentrations of H_2_O_2_. Cyclic voltammograms were registered at 0.05 V/s; Figure S2: Cyclic voltammograms of differently modified GR electrodes in the absence (dashed line) and in the presence (solid line) of 29.4 mM H_2_O_2_ (a) and current responses to H_2_O_2_ of differently modified GR electrodes registered by CPA (b) in 0.05 M SA buffer with 0.1 M KCl. Cyclic voltammograms were registered at 0.05 V/s, and CPA responses at −0.35 V vs. Ag/AgCl_(3 M KCl)_; Figure S3: The cyclic voltammograms of glucose biosensors in the presence (a) and absence (b) of O_2_. Cyclic voltammograms were registered in 0.05 M SA buffer with 0.1 M KCl, at 0.05 V/s, 60 min of deaeration by argon (b); Figure S4: The repeatability study of biosensors based on GR/DGNS/Cys/GOx or GR/DGNS/Cys/PANI-AuNPs-GOx/GOx electrodes. Current responses of CPA were registered in 0.05 M SA buffer with 0.1 M KCl and 0.50 mM glucose at −0.35 V vs. Ag/AgCl_(3 M KCl)_; Figure S5: The influence of interfering species on the current response of glucose biosensors based on GR/DGNS/Cys/GOx (a) and GR/DGNS/Cys/PANI-AuNPs-GOx/GOx (b) electrodes. The measurements of CPA were performed at −0.35 V vs. Ag/AgCl_(3 M KCl)_ in a 10-fold diluted sample of blood serum after the addition of 0.5 mM glucose, 1.0 mM fructose, mannose, xylose, saccharose, or galactose, and finally 2.0 mM glucose; Figure S6: The determination of 0.420 mM glucose using the GR/DGNS/Cys/PANI-AuNPs-GOx/GOx electrode. The CPA measurements were performed at −0.35 V vs. Ag/AgCl_(3 M KCl)_ in a 10-fold diluted sample of blood serum using the addition method.

## Abbreviations

The following abbreviations are used in this manuscript:

GR	Graphite rod
DGNS	Dendritic gold nanostructures
Cys	Cystamine
SAM	Self-assembled monolayer
GOx	Glucose oxidase
PANI	Polyaniline
AuNPs	Gold nanoparticles
LR	Linear range
LOD	Limit of detection
H_2_O_2_	Hydrogen peroxide
DET	Direct electron transfer
O_2_	Oxygen
GOx(FAD)	Oxidized form of the enzyme
GOx(FADH_2_)	Reduced form of glucose oxidase
Fe@Au	Gold-coated magnetic iron oxide nanoparticles
AuNC	Gold nanocluster
NPAu	Nanoporous gold
PtNPs	Platinum nanoparticles
RGO-Fe_3_O_4_	Reduced graphene oxide–magnetic nanoparticles
Ppy	Polypyrrole
GC	Glassy carbon
Au@ILs-polysome	Gold nanoparticles and ionic liquids-based polysome
Au	Gold
K_4_[Fe(CN)_6_]·3H_2_O	Potassium hexacyanoferrate(II) trihydrate
HAuCl_4_·3 H_2_O	Tetrachloroauric acid trihydrate
CH_3_COONa·3H_2_O	Sodium acetate trihydrate
KCl	Potassium chloride
SA	Sodium acetate
Al_2_O_3_	α-Aluminium oxide
KNO_3_	Potassium nitrate
K_3_[Fe(CN)_6_]	Potassium hexacyanoferrate(III)
GA	Glutaraldehyde
AA	L-ascorbic acid
UA	Uric acid
CPA	Constant potential amperometry
CV	Cyclic voltammetry
Pt	Platinum
Ag/AgCl_(3 M KCl)_	Reference electrode
*R*^2^	Determination coefficient
Δ*I*_max_	Maximal current
*K*_M(app)_	Michaelis constant
EASA	Electroactive surface area
*I*_p_	Maximal peak current
n	Number of electrons appearing in the half-reaction
*D*	Diffusion coefficient
*C*	Concentration of electroactive species
*v*	Potential scan rate
Cu-nanoflower	Copper nanoflower
PVA	Polyvinyl alcohol
GO NFs	Graphene oxide nanofibers
HRP	Horseradish peroxidase
AuNNs	Gold nanopine needles
BSA	Bull serum albumin
PEGDE	Poly(ethylene glycol) diglycidylether
DENPs	Dualenzyme nanoparticles
CP	Carbon paste
OOPpy	Overoxidized polypyrrole
RSD	Relative standard deviation
PMS	Phenazine methosulfate
PD	1,10-Phentroline-5,6-dione
