# Supplementary material for: A Platform for the Glucose Biosensor Based on Dendritic Gold Nanostructures and Polyaniline-Gold Nanoparticles Nanocomposite
Source: Biosensors (Basel). 2025 Mar 19;15(3):196. doi: 10.3390/bios15030196 (PMC11940116; doi:10.3390/bios15030196)
Supplement: Supplementary file 1 [file biosensors-15-00196-s001.zip › biosensors-3434534-supplementary.pdf]

A Platform for the Glucose Biosensor Based on Dendritic Gold Nanostructures and Polyaniline-Gold Nanoparticles Nanocomposite

Natalija German [1]*, Anton Popov [1,2], Arunas Ramanavicius [2], Almira Ramanaviciene [1,2]*

[1] Department of Immunology and Bioelectrochemistry, State Research Institute Centre for Innovative Medicine, Santariskiu 5, LT-08406, Vilnius, Lithuania; anton.popov@imcentras.lt (A.P)

[2] NanoTechnas – Center of Nanotechnology and Materials Science, Faculty of Chemistry and Geosciences, Vilnius University, LT-03225, Vilnius, Lithuania ; arunas.ramanavicius@chf.vu.lt (A.R.)

*Correspondence: natalija.german@imcentras.lt (N.G.), almira.ramanaviciene@chf.vu.lt (A.R.)

Supplementary Materials

The synthesis of 6 nm gold nanoparticles and PANI-AuNPs-GOx nanocomposites

To synthesize 6 nm AuNPs, 80 mL of 0.0125% [w/v] $HAuCl_4$ solution and 20 mL of a solution containing 4 mL 1% [w/v] trisodium citrate and 0.5 mL of 1% [w/v] of tannic acid were heated to +60 °C on a magnetic stirrer. After that, both solutions were mixed, heated up to +98 °C, stirring, and kept at this temperature for 3 min to yield a red-wine-coloured solution. Then, the Erlenmeyer flask with 6 nm AuNPs was transferred into the ice bath.

A two-days enzyme-assisted synthesis of PANI-AuNPs-GOx nanocomposites was performed in 0.05 M SA buffer, pH 5.6, containing 0.05 M of glucose, 0.75 mg/mL GOx and 0.50 M of aniline in the presence of 0.46×10^{16} particles/L of 6 nm AuNPs at room temperature (+20 ± 2°C) in darkness. Formed polymer nanocomposites were separated from the polymerization solution by centrifugation using IEC CL31R Multispeed centrifuge from Thermo Electron Industries S.A.S. (Château-Contier-sur-Mayenne, France) for 8 min (14.6×10^3 ×g). After that, PANI-AuNPs-GOx nanocomposites were washed two times with SA buffer and centrifuged.

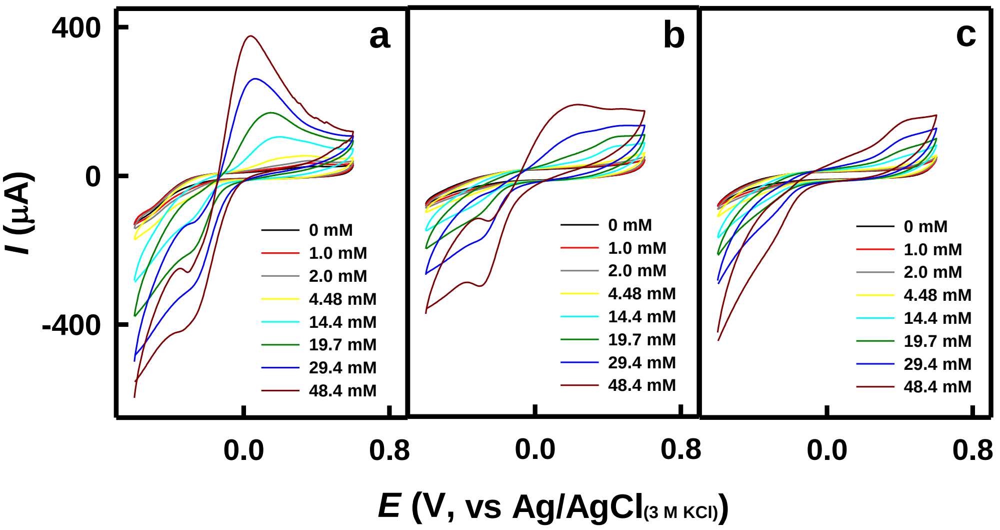

Figure S1. Cyclic voltammetric measurements of GR/DGNS/Cys (**a**), GR/DGNS/Cys/PANI-AuNPs-GOx/GOx (**b**), and GR/DGNS/Cys/GOx (**c**) electrodes in 0.05 M SA buffer with 0.1 M KCl containing various concentrations of H_2O_2. Cyclic voltammograms were registered at 0.05 V/s.

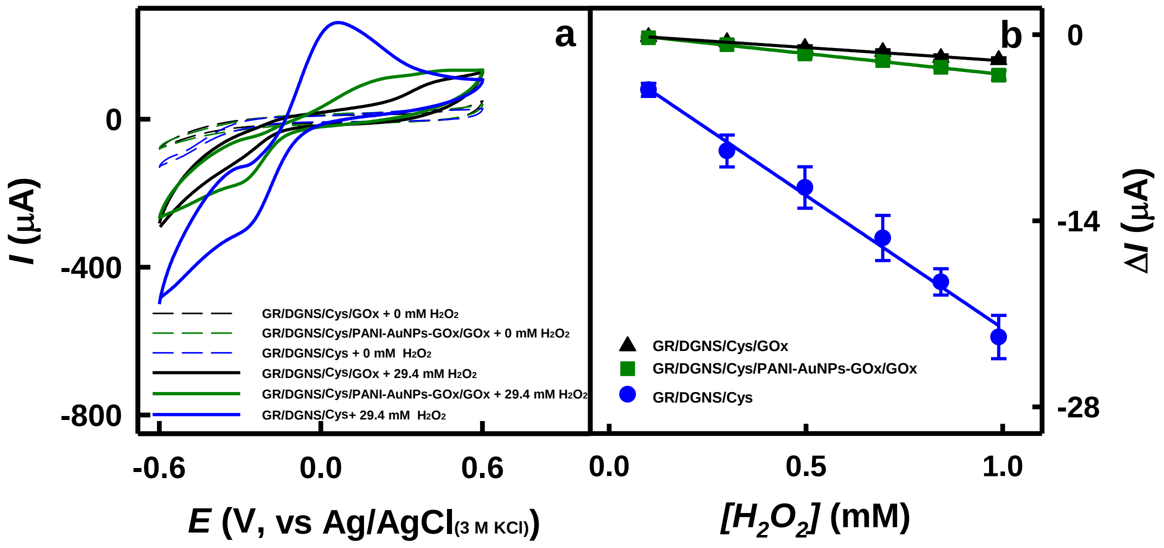

Figure S2. Cyclic voltammograms of differently modified GR electrodes in the absence (dashed line) and in the presence (solid line) of 29.4 mM H_2O_2 (**a**) and current responses to H_2O_2 of differently modified GR electrodes registered by CPA (**b**) in 0.05 M SA buffer with 0.1 M KCl. Cyclic voltammograms were registered at 0.05 V/s, and CPA responses at –0.35 V vs. Ag/AgCl(3 M KCl).

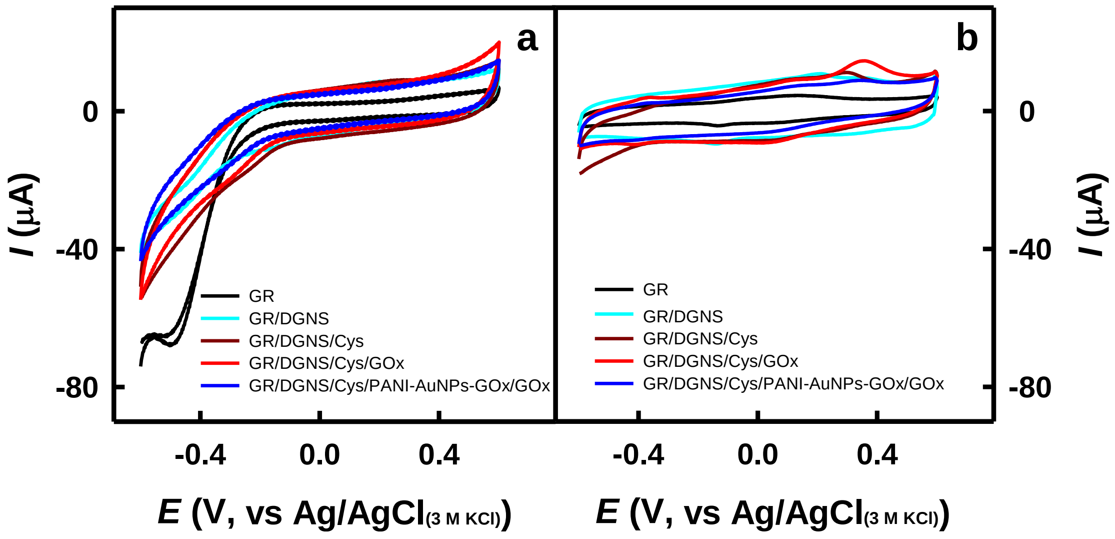

Figure S3. The cyclic voltammograms of glucose biosensors in the presence (**a**) and absence (**b**) of O_2. Cyclic voltammograms were registered in 0.05 M SA buffer with 0.1 M KCl, at 0.05 V/s, 60 min of deaeration by argon **(b)**.

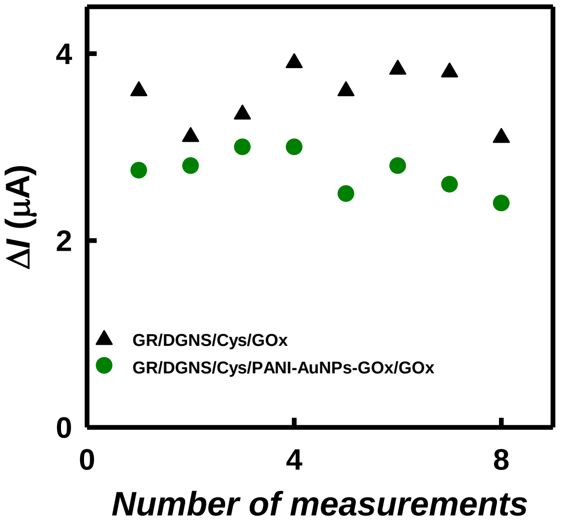

Figure S4. The repeatability study of biosensors based on GR/DGNS/Cys/GOx or GR/DGNS/Cys/PANI-AuNPs-GOx/GOx electrodes. Current responses of CPA were registered in 0.05 M SA buffer with 0.1 M KCl and 0.50 mM glucose at –0.35 V vs. Ag/AgCl(3 M KCl).

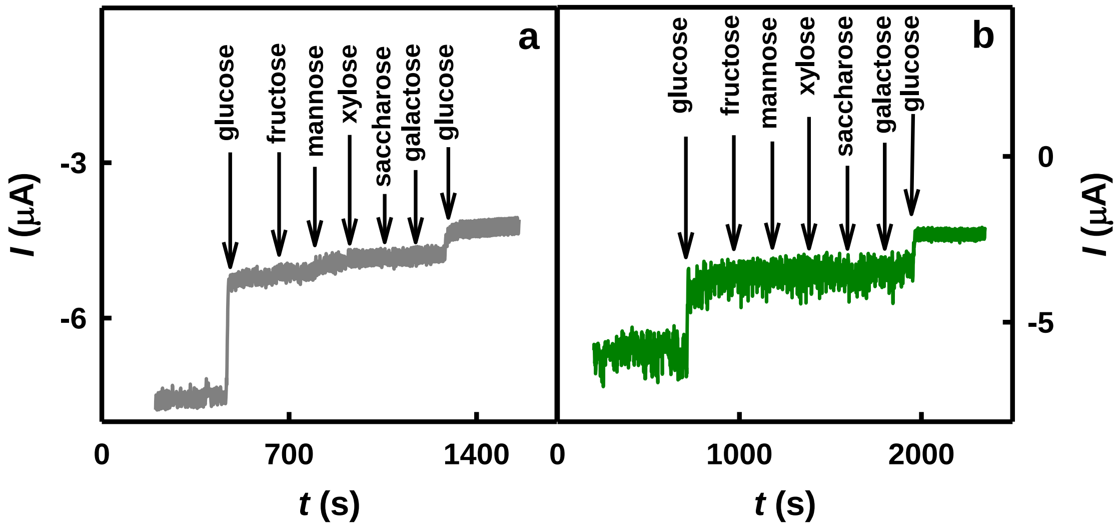

Figure S5. The influence of interfering species on the current response of glucose biosensors based on GR/DGNS/Cys/GOx (**a**) and GR/DGNS/Cys/PANI-AuNPs-GOx/GOx (**b**) electrodes. The measurements of CPA were performed at –0.35 V vs. Ag/AgCl(3M KCl) in a 10-fold diluted sample of blood serum after the addition of 0.5 mM glucose, 1.0 mM fructose, mannose, xylose, saccharose, or galactose, and finally 2.0 mM glucose .

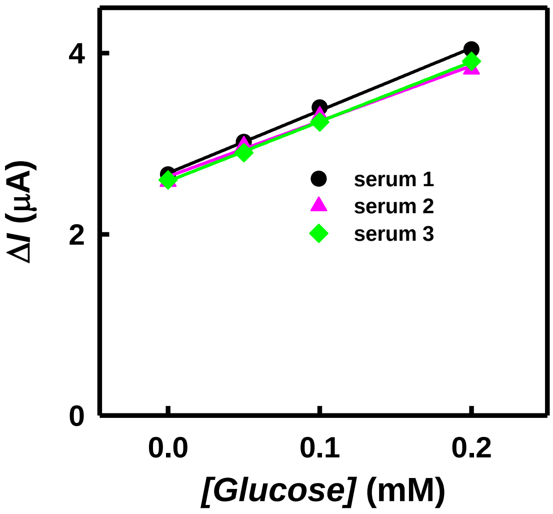

Figure S6. The determination of 0.420 mM glucose using the GR/DGNS/Cys/PANI-AuNPs-GOx/GOx electrode. The CPA measurements were performed at –0.35 V vs. Ag/AgCl$_{(3M\ KCl)}$ in a 10-fold diluted sample of blood serum using the addition method.
